# Lysyl oxidase inhibits BMP9-induced osteoblastic differentiation through reducing Wnt/β-catenin via HIF-1a repression in 3T3-L1 cells

**DOI:** 10.1186/s13018-023-04251-0

**Published:** 2023-11-29

**Authors:** Jie Zhang, FangLin Ye, AiHua Ye, BaiCheng He

**Affiliations:** 1 Department of Pharmacology, School of Pharmacy, Chongqing Medical University, No. 1 Yixueyuan Road, Yuzhong, Chongqing, 400016 People’s Republic of China; 2 Key Laboratory of Biochemistry and Molecular Pharmacology of Chongqing, Chongqing Medical University, Chongqing, 400016 People’s Republic of China

**Keywords:** Osteogenic differentiation, Lysyl oxidase, Bone morphogenetic protein 9, Hypoxia-inducible factor 1α, Wnt/β-catenin

## Abstract

**Background:**

Bone morphogenetic protein 9 (BMP9) is a promising growth factor in bone tissue engineering, while the detailed molecular mechanism underlying BMP9-oriented osteogenesis remains unclear. In this study, we investigated the effect of lysyl oxidase (Lox) on the BMP9 osteogenic potential via in vivo and in vitro experiments, as well as the underlying mechanism.

**Methods:**

PCR assay, western blot analysis, histochemical staining, and immunofluorescence assay were used to quantify the osteogenic markers level, as well as the possible mechanism. The mouse ectopic osteogenesis assay was used to assess the impact of Lox on BMP9-induced bone formation.

**Results:**

Our findings suggested that Lox was obviously upregulated by BMP9 in 3T3-L1 cells. BMP9-induced Runx2, OPN, and mineralization were all enhanced by Lox inhibition or knockdown, while Lox overexpression reduced their expression. Additionally, the BMP9-induced adipogenic makers were repressed by Lox inhibition. Inhibition of Lox resulted in an increase in c-Myc mRNA and β-catenin protein levels. However, the increase in BMP9-induced osteoblastic biomarkers caused by Lox inhibition was obviously reduced when β-catenin knockdown. BMP9 upregulated HIF-1α expression, which was further enhanced by Lox inhibition or knockdown, but reversed by Lox overexpression. Lox knockdown or HIF-1α overexpression increased BMP9-induced bone formation, although the enhancement caused by Lox knockdown was largely diminished when HIF-1α was knocked down. Lox inhibition increased β-catenin levels and decreased SOST levels, which were almost reversed by HIF-1α knockdown.

**Conclusion:**

Lox may reduce the BMP9 osteoblastic potential by inhibiting Wnt/β-catenin signaling via repressing the expression HIF-1α partially.

**Graphical abstract:**

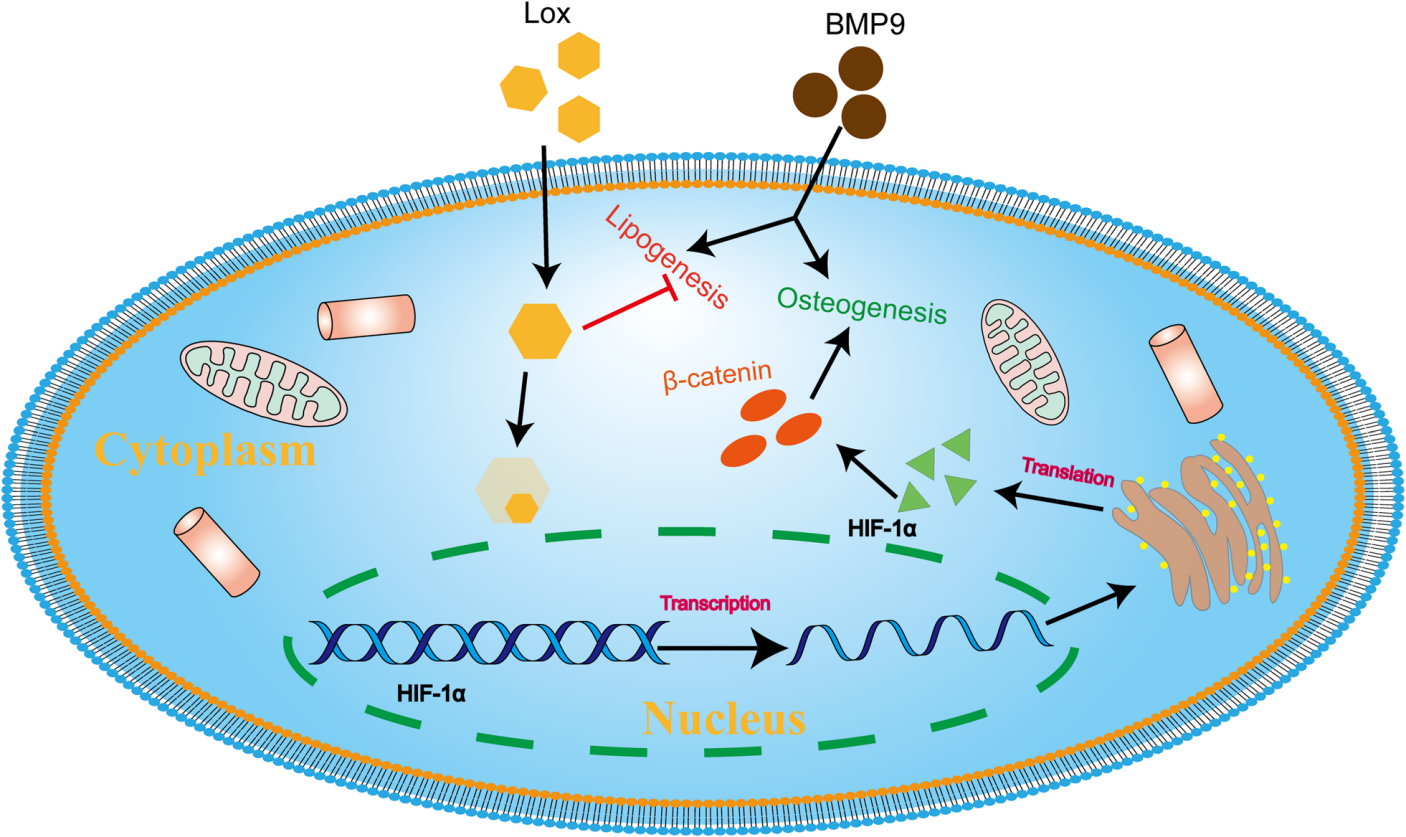

## Introduction

Depending on the context or particular inducer, multipotent stem cells can be committed to various lineages like osteoblastic, chondrogenic, adipogenic, or myogenic [1]. Osteogenesis keeps intimate connection with adipogenesis, since the increase of osteogenesis may be based on the cost of adipogenesis, and vice versa. Several members of bone morphogenetic protein (BMP) possess excellent potential to commit progenitor cells to osteoblastic lineage, such as BMP2, BMP7, and BMP9 [2, 3]. BMP9 is one of the most strong osteogenic factors, which could be a potent substitute for bone tissue engineering [4, 5]. However, adipocytes are evidently present in the BMP9-induced bone masses [6]. Thus, the BMP9 osteogenic potential might be strengthened by reducing the BMP9-induced adipogenesis.

Several critical factors are related with boosting adipogenic fate decision in stem cells, such as peroxisome proliferator-activated receptor gamma (PPARγ) and C/EBP-α [7]. PPARγ plays an important role in regulating BMP9-induced osteogenesis. Our earlier research discovered that all-trans-retinoic acid may increase the osteogenic potential of BMP9 and diminish its capacity to induce adipogenesis by inhibiting PPARγ expression [8].

Lysyl oxidase (Lox) is a copper-dependent enzyme, which is involved in the metabolism of lysine or lysine residues by turning them into active products that are then used to cross-link extracellular proteins, such as aldehydes [9]. This process might be very crucial for the collagen fibrils stabilization of or elastin elasticity [10]. Thus, Lox plays a very important role in development, such as skin and respiratory system [11]. Function loss or overexpression of Lox is implicated with numerous illnesses, such as myelofibrosis, lung cancer, and metastasis of cancer cells [12, 13]. In addition, Lox also plays a crucial role in managing cell proliferation and differentiation, including regulating the adipocyte formation from stem cells [14]. Therefore, Lox might be a target to enhance the BMP9 osteoblastic potential. However, it remains unknown whether the BMP9-induced osteogenic differentiation is associated with Lox.

In this study, we investigated the effect of BMP9 on Lox in multipotent stem cells, and the effect of Lox on the BMP9 osteogenic potential, as well as the underlying putative molecular mechanism. Our study might offer another strategy to increase the BMP9-based osteogenesis for bone tissue engineering.

## Materials and methods

### Cell culture and chemicals

C3H10T1/2, C2C12, 3T3L-1, and HEK-293 cell lines were purchased from the American Type Culture Collection. Mouse embryonic fibroblasts (MEFs) were extracted from embryos of NIH mouse and donated by the Molecular Oncology Laboratory of Chicago Medical Center [15]. β-aminopropionitrile (BAPN, #T13475) was purchased from Topscience (Shanghai, China). Cells were cultured in complete DMEM medium (containing 10% fetal bovine serum, 100 kU/L penicillin and 0.1 g/L streptomycin) at 37 °C with 5% CO_2_. Primary antibodies against GAPDH (10,494–1-AP) and sclerostin (21,933–1-AP) were ordered from Proteintech (China branch); BMP9 (sc-514211), Lox (sc-32409), Runx2 (sc-390351), OPN (sc-21742), β-catenin (sc-7963), H_2_A_x_ (sc-517336), and HIF-1α (sc-13515) were ordered from Santa Cruz Biotechnology (China branch).

### Construction of recombinant adenovirus vector

Recombinant adenovirus vectors for this study was constructed using AdEasy system [16, 17]. Briefly, coding sequence of BMP9, Lox, HIF-1α, and green fluorescent protein (GFP) were amplified with PCR; siRNA oligos of Lox, HIF-1α, and β-catenin were synthesized commercially. All these products were cloned into the shuttle plasmids separately. Then, homologous recombination is carried out between the linearized shuttle plasmid and the adenovirus skeleton plasmid. Finally, the correct products are linearized and transfected into HEK-293 cells for recombinant adenoviruses package. The adenovirus vectors were tagged with GFP or red fluorescent protein (RFP) to track virus, and termed as AdBMP9, AdLox, AdHIF-1α, AdsiLox, AdsiHIF-1α, or AdsiBC (siRNA for β-catenin). Recombinant adenovirus expressing GFP (AdGFP) only was used as vehicle control.

### Mineral deposition assay

After cells were treated with AdBMP9, AdsiLox, AdLox, AdBMP9 plus AdHIF-1α, AdBMP9 plus AdsiLox, and other reagents for 48 h, the medium was replaced with osteogenic induction medium [18]. On day 20, the growth media was removed, and the cells were gently washed with phosphate-buffered saline (PBS), fixed with 0.05% glutaraldehyde for 10 min, and then rinsed once more in PBS (pH 4.2). After that, cells were stained for 5 min with Alizarin red S working solution (0.4%). Finally, discarded the Alizarin red S solution and washed the cells with PBS (pH 4.2) for three times. Under a microscopy (IX53, Olympus), photographs were captured after scanning the plates.

### Total RNA extraction, reverse transcription (RT) and polymerase chain reaction (PCR)

Total RNAs were extracted with Trizol reagent, and the complimentary DNA was produced with RT following the introduction of kit (R037A, Takara). Then, the product was diluted 5 to 10 folds and used as template for PCR assay. Real-time PCR assay was performed with SYBR green kit and CFX Connect system (Bio-Rad, USA). The relative mRNA expression level was calculated using 2^−ΔΔCt^ method, and normalized with the glyceraldehyde triphosphate dehydrogenase (GAPDH) mRNA level. The primers used in this experiment are shown in Table 1.

**Table 1:** The primers used for PCR assay

Gene	Primer	Sequence (5′ → 3′)
Lox	F	TGCCAACACACAGAGGAGAG
	R	CCAGGTAGCTGGGGTTTACA
C/EBP-α	F	GAGGGGAGGGACTTAGGTGT
	R	TGCCCCCATTCTCCATGAAC
PPARγ	F	TTTTCAAGGGTGCCAGTTTC
	R	AATCCTTGGCCCTCTGAGAT
HIF-1α	F	CTGGGACTTTCTTTTACCATGC
	R	AATGGATTCTTTGCCTCTGTGT
GAPDH	F	ACCCAGAAGACTGTGGATGG
	R	CACATTGGGGGTAGGAACAC

*F* Forward; *R* Reverse

### Western blot analysis

Cells were seeded in the six-well plate and treated with AdBMP9, AdsiLox, AdLox, AdBMP9 plus AdHIF-1α, AdBMP9 plus AdsiLox, and other reagents. Proteins were extracted at the final stage of the experiment using radio immunoprecipitation assay (RIPA) lysis buffer (#R0020-100 Solarbio, China). Target proteins were separated using sodium dodecyl sulfate polyacrylamide gel, then transferred to polyvinylidene difluoride membrane, blocked with 5% bovine serum albumin for 1 h at room temperature, probed with the corresponding primary anti-body at 4 °C for 2 h, and incubated with horseradish peroxidase-labeled secondary antibody at 4 °C for 30 min. Finally, chemiluminescent kit (160,072, Saimike Biotech, Chongqing, China) was used to show the protein, and data were obtained using a Bio-Rad gel imager (ChemiScope 6200, Qinxiang, Shanghai China).

### Immunofluorescence assay

Cells were fixed with 4% paraformaldehyde for 20 min, washed with PBS; treated with 0.5% Triton X-100 for 20 min, washed with PBS; blocked with goat serum at 37 °C for 30 min, then incubated with primary antibody at 4 °C overnight. Cells were washed with PBS and incubated with fluorescent secondary antibody (#A23410-1, Abbkine) at 37 °C for 30 min, washed three times with PBS, and incubated with 2-(4-amidinophenyl) -6-indolecarbamidine dihydrochloride for 10 min. Finally, images were obtained using microscope (IX53, Olympus).

### Mouse ectopic osteogenesis assay

Nude mice were ordered from the Experimental Animal Center of Chongqing Medical University (five per group, 6-week-old, females, and body weight is 20–24 g). This experiment was approved by the Institutional Animal Care and Use Committee of Chongqing Medical University (Chongqing, China). Cells were cultured in 100 mm dishes, and pre-treated with AdBMP9, AdBMP9 plus AdsiLox, AdBMP9 plus AdHIF-1α, or AdBMP9 plus AdsiLox and AdsiHIF-1α. After 36 h, cells were collected, centrifuged and resuspended with PBS (4 °C). Finally, cells were implanted to mice flanks subcutaneously (5 × 10^6^ cells per injection). After 4 weeks, the nude mice were euthanized and the bone tissues were collected for imaging and histological evaluation.

### Micro-computed tomographic (μ-CT) analysis

Bone masses were scanned with micro-CT (SCANCO Medical AG, Switzerland). The data were analyzed with μ-CT 516.1 (provided by the scanner manufacture) for three-dimensional reconstruction and quantification.

### Tissue section preparation, staining, and evaluation

The retrieved bone samples were fixed with 4% paraformaldehyde for 48 h, and decalcified using ethylenediaminetetraacetic acid decalcification solution for 2 weeks. Then, specimens were embedded with paraffin for section preparation. Finally, the sections were subjected to hematoxylin and eosin stain, and images were obtained using microscope (Ci-L, Nikon).

### Statistical analysis

Data were analyzed using software GraphPad Prism 6 and shown as mean ± standard deviation. Student *t*-test was used to analyze the difference between two groups. Each assay was repeated at least three times independently. The difference was defined as statistical significance if the *P* value less than 0.05.

## Results

### BMP9 up-regulates lox expression in multipotent stem cells

**Fig. 1:**
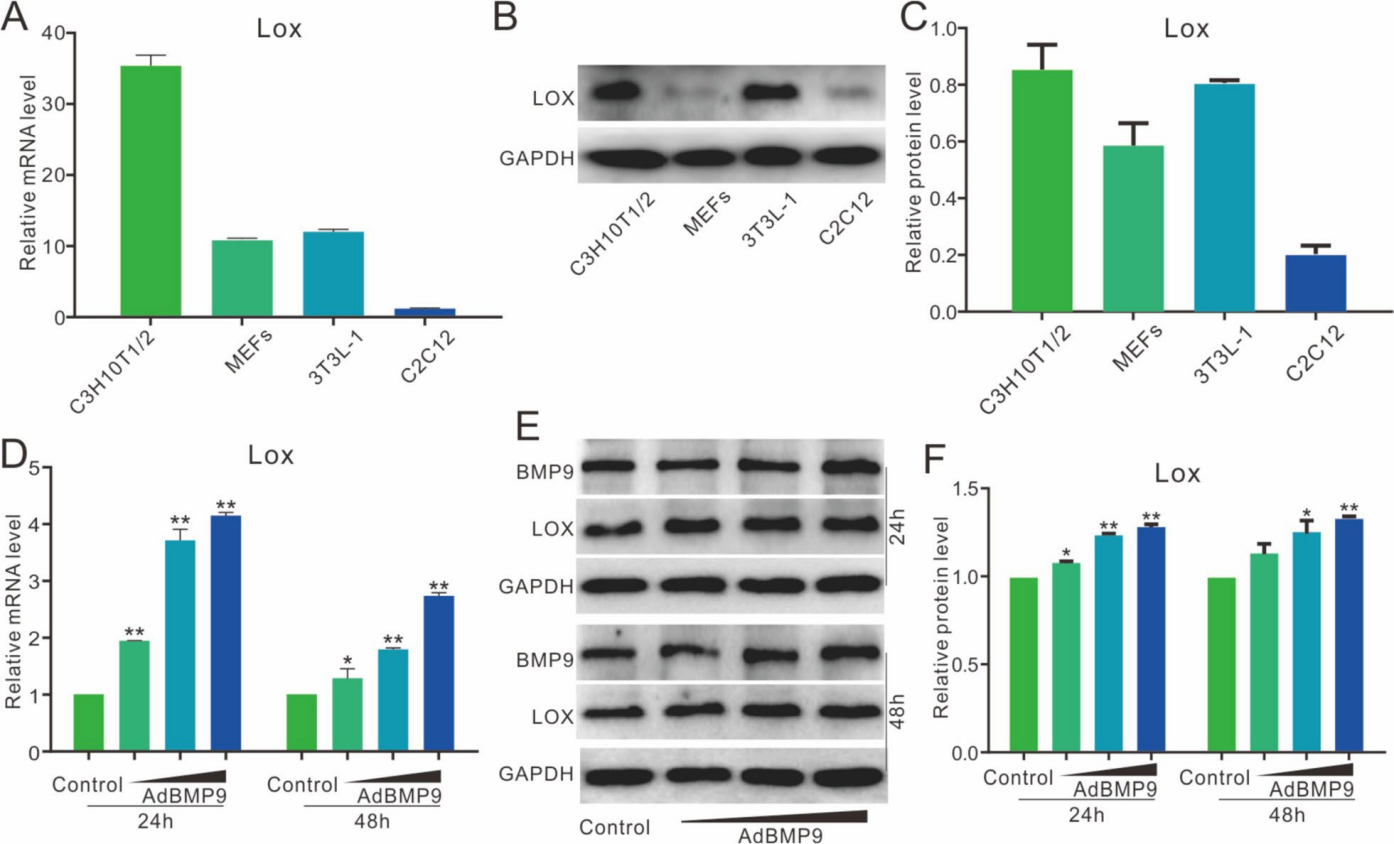
BMP9 up-regulates expression of lox in MPCs **A** Real-time PCR analysis results show the endogenous mRNA level of Lox in C3H10T1/2, MEFs, 3T3-L1, and C2C12 cells. **B**, **C** Western blotting assay results show the endogenous protein level of Lox in C3H10T1/2, MEFs, 3T3-L1, and C2C12 cells. **D** Real-time PCR assay results show the effect of BMP9 on Lox in 3T3-L1 cells. **E**, **F** Western blotting analysis results show the effect of BMP9 on Lox in 3T3-L1 cells. (*p < 0.05, **p < 0.01 vs. control)

Real-time PCR assay results indicated that the Lox mRNA is detectable in these multipotent stem cells (Fig. 1A). Besides, the Lox protein level was greater in C3H10T1/2 and 3T3-L1 cells (Fig. 1B, C). As a pre-adipocyte cell line, 3T3-L1 is an excellent cell model for analyzing the balance between adipogenic and osteogenic differentiation. The Lox mRNA and protein level were improved greatly by BMP9 in 3T3-L1 cells (Fig. 1D–F). These results suggested that Lox may be involved in regulating BMP9 osteogenic capability in multipotent stem cells.

### Effects of Lox on BMP9-induced osteogenic and adipogenic markers in 3T3-L1 cells

**Fig. 2:**
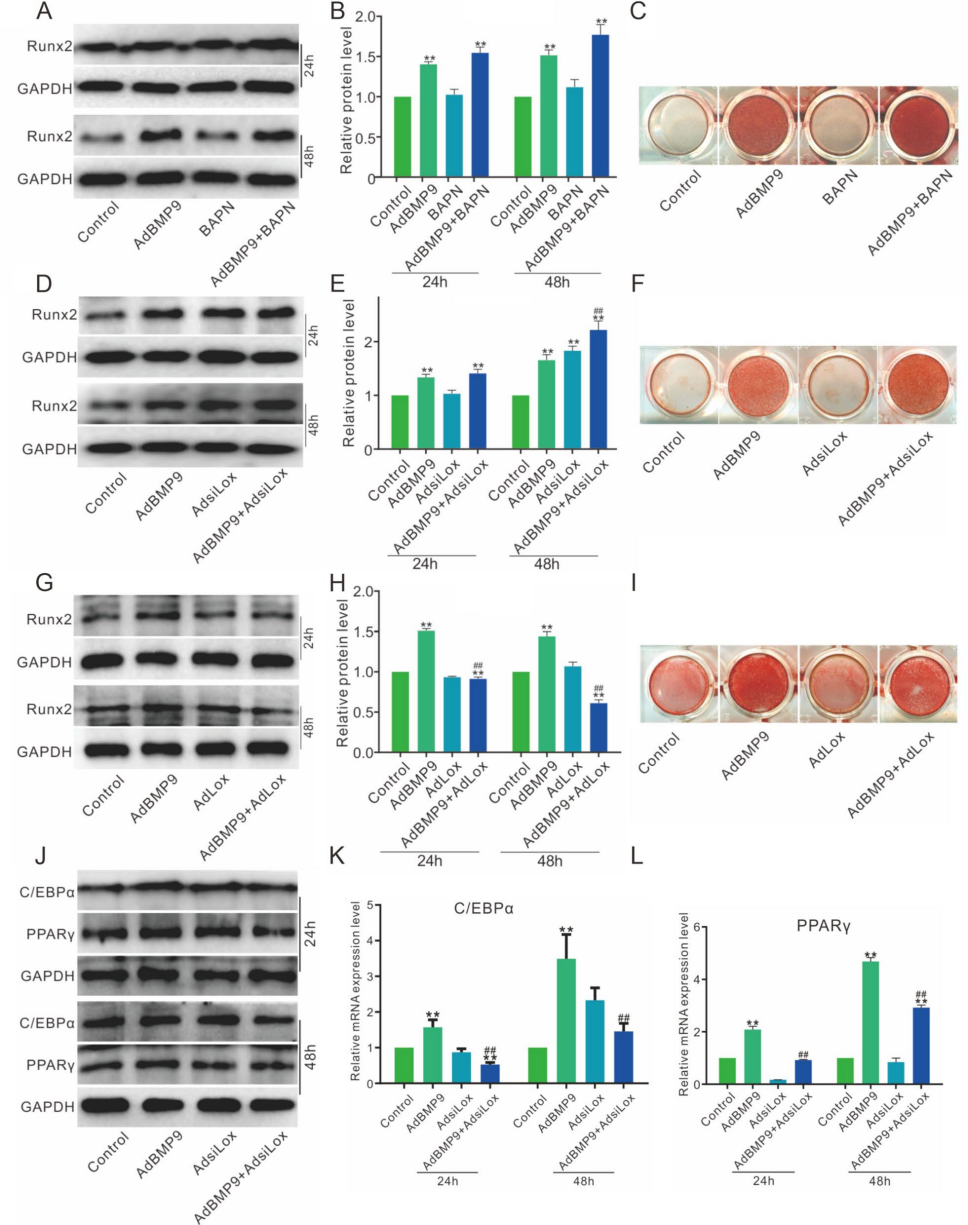
Effects of Lox on the osteogenic and adipogenic markers induced by BMP9 in 3T3-L1 cells **A**, **B** Effect of BMP9 and BAPN on the Runx2 protein level. (**C**) The effect of BMP9 and BAPN on the matrix mineralization. **D**, **E** Effect of BMP9 and Lox knockdown on the Runx2 protein level. **F** Effect of BMP9 and Lox knockdown on the matrix mineralization. **G**, **H** The BMP9 and Lox effect on the Runx2 protein level. **I** The effect of BMP9 and Lox on matrix mineralization. **J** Effect of BMP9 and Lox knockdown on the protein level of C/EBPα or PPARγ. **K**, **L** BMP9 and Lox knockdown effect on the mRNA level of C/EBPα or PPARγ. (BAPN: β-aminopropionitrile, inhibitor of Lox, the concentration is 200 μM. **p < 0.01 vs. control; #*p* < 0.05, ##*p* < 0.01 vs. BMP9 treated group)

Western blot results showed that BMP9 increased Runx2 level, which were substantially enhanced by Lox inhibition (Fig. 2A, B). The BMP9-induced mineralization was also raised by Lox inhibition (Fig. 2C). In addition, the BMP9-induced Runx2 protein level was increased by Lox knockdown (Fig. 2D, E), as well as the mineralization (Fig. 2F). However, Lox overexpression inhibited the BMP9-induced Runx2 and mineralization (Fig. 2G-I). BMP9 increased the PPARγ and C/EBP-α protein expression, which were suppressed by Lox knockdown (Fig. 2J). Similar results were attained in mRNA expression (Fig. 2K, L). These data indicated that the improvement of BMP9 osteogenic-induction potential by Lox knockdown may be realized through the reduce of adipogenic differentiation partly in 3T3-L1 cells.

### Effects of Wnt/β-catenin on the osteoblastic markers affected by BMP9 and Lox inhibition in 3T3-L1 cells

**Fig. 3:**
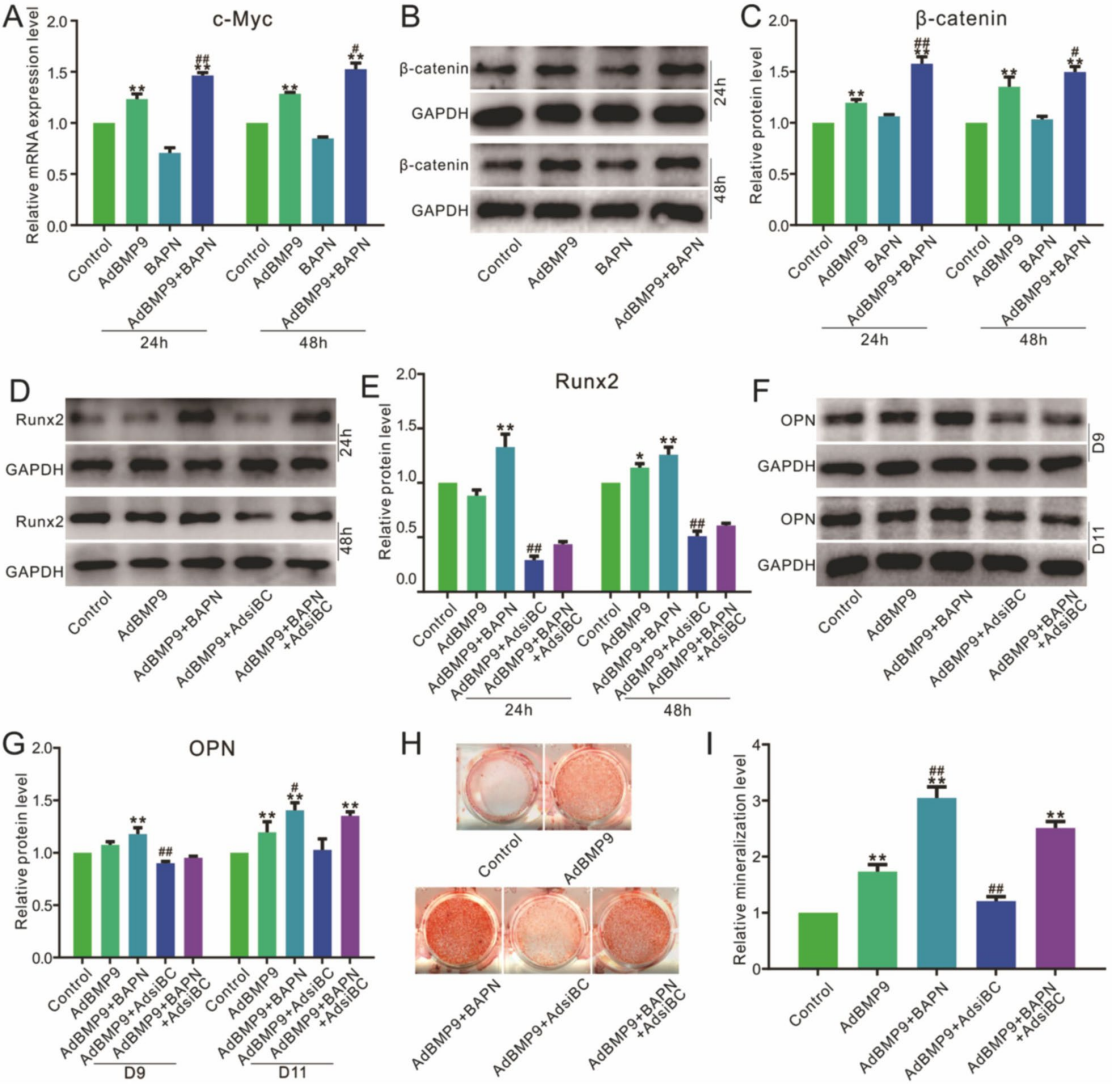
Effects of Wnt/β-catenin on the osteoblastic markers, which affected by BMP9 and Lox specific inhibitor in 3T3-L1 cells. **A** Effect of BAPN on the mRNA level of c-Myc induced by BMP9. **B**, **C** Effect of BAPN on the protein level of β-catenin induced by BMP9. **D**, **E** Effect of BAPN and siRNA of β-catenin on the protein level of Runx2 induced by BMP9. **F**, **G** Effect of BAPN, and siRNA of β-catenin on the protein level of OPN induced by BMP9. **H**, **I** Effect of BAPN, and siRNA of β-catenin on the mineralization induced by BMP9. (BAPN: β-aminopropionitrile, inhibitor of Lox, the concentration is 200 μM. *p < 0.05, **p < 0.01 vs. control; #*p* < 0.01, ##*p* < 0.01 vs. BMP9 treated group)

Real-time PCR results demonstrated that BMP9 increased c-Myc mRNA level, which was reduced by Lox inhibition; the c-Myc mRNA was increased greatly when BMP9 combined with Lox inhibition (Fig. 3A). Western blot assay showed the β-catenin protein level was increased by BMP9; Lox inhibition exhibited no substantial effect on β-catenin protein level, but it was increased obviously by combining with BMP9 (Fig. 3B, C). BMP9-induced Runx2 and OPN, and the mineralization were increased by Lox inhibition, which was reduced by combining with β-catenin knockdown (Fig. 3D–I). These results suggested that Lox may inhibit the BMP9 osteogenic-induction potential through decreasing Wnt/β-catenin signaling activity.

### Effects of BMP9 and lox on HIF-1α in 3T3-L1 cells

**Fig. 4:**
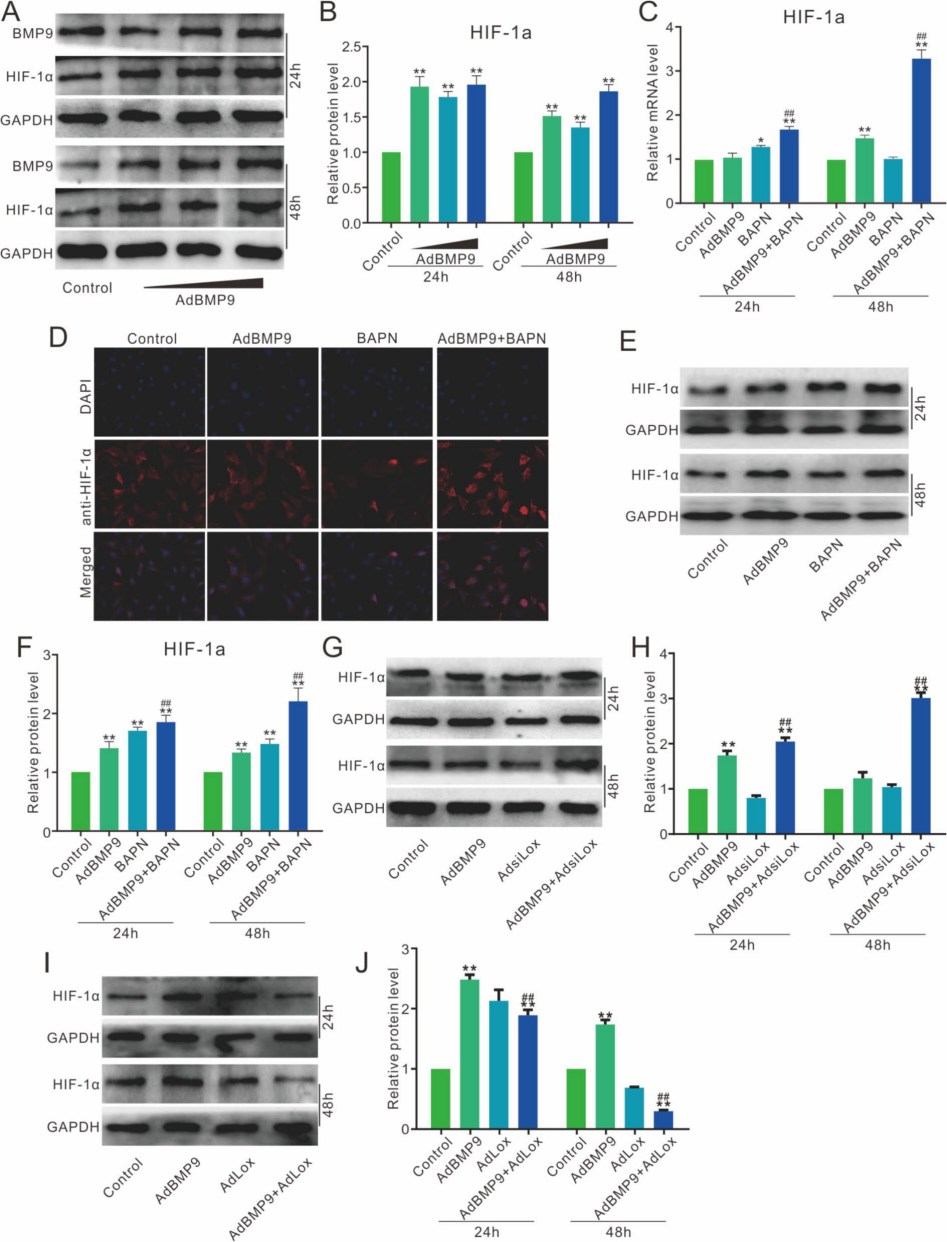
Effects of BMP9 and Lox on HIF-1α in 3T3-L1 cells **A**, **B** Effect of BMP9 on the protein level of HIF-1α. **C** Effect of BAPN on the mRNA level of HIF-1α induced by BMP9. **D** Effect of BAPN on the protein level of HIF-1α induced by BMP9. **E**, **F** Effect of BAPN on the protein level of HIF-1α induced by BMP9. **G**, **H** Effect of siRNA of Lox on the protein level of HIF-1α induced by BMP9. **I**, **J** Effect of Lox on the protein level of HIF-1α induced by BMP9. (BAPN: β-aminopropionitrile, inhibitor of Lox, the concentration is 200 μM. *p < 0.05, **p < 0.01 vs. control; ##*p* < 0.01 vs. BMP9 treated group)

Western blot assay showed the HIF-1α protein level was markedly increased by BMP9 in 3T3-L1 cells (Fig. 4A and B). Real-time PCR results showed the effect of BMP9 on increasing HIF-1α mRNA was enhanced by Lox inhibition (Fig. 4C). Immunofluorescent stain showed that the BMP9-induced HIF-1α was promoted by Lox inhibition (Fig. 4D). Western blot assay showed that the BMP9-induced HIF-1α was enhanced by Lox inhibition (Fig. 4E and F). The BMP9-induced HIF-1α was strengthened by Lox knockdown (Fig. 4G and H), but decreased obviously by Lox over-expression (Fig. 4I and J). These results suggested that HIF-1α may be associated with the effect of Lox on the BMP9 osteogenic ability.

### Effects of lox and HIF-1α on BMP9-induced bone formation in 3T3-L1 cells

**Fig. 5:**
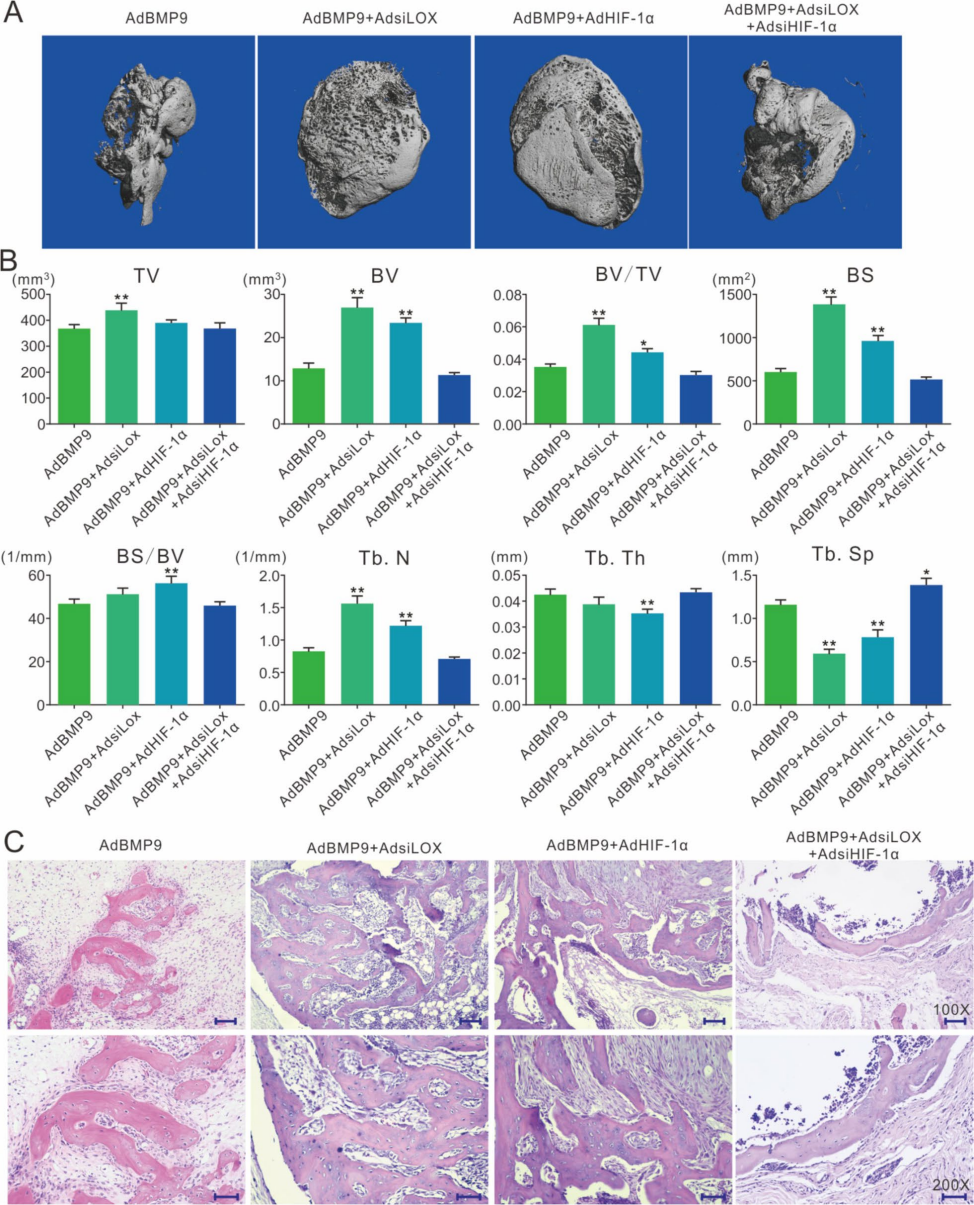
Effects of Lox and/or HIF-1α on the ectopic bone formation induced by BMP9 in 3T3-L1 cells **A** Effect of BMP9, siRNA of Lox, HIF-1α, and/or siRNA of HIF-1α on bone formation. **B** Effect of BMP9, Lox knockdown, HIF-1α and/or HIF-1α knockdown on bone formation in 3T3-L1 cells (TV: total volume, BV bone volume, BS: bone surface area, Tb.N: trabecular number, Tb.Th: trabecular thickness, Tb.Sp: trabecular separation). **C** H&E staining show the effect of BMP9, Lox knockdown, HIF-1α and/or HIF-1α knockdown on bone formation in 3T3-L1 cells (Scale bar: 200 μm for upper panel, 50 μm for lower panel. *p < 0.05, **p < 0.01 vs. group treated with BMP9

The 3D reconstruction of μ-CT imaging showed that the osteogenic potential of BMP9 was increased by Lox knockdown or HIF-1α overexpression, but the effect of Lox knockdown on promoting BMP9 osteogenic potential was partly inhibited by HIF-1α knockdown (Fig. 5A). The quantification analysis of the μ-CT scanning showed the similar results (Fig. 5B). H&E stain showed that trabecular bone induced by BMP9 was increased by Lox knockdown, which was attenuated by HIF-1α knockdown (Fig. 5C). These results suggested that the increase of BMP9 osteoblastic potential by Lox knockdown may be partially mediated through HIF-1α upregulation.

### Effects of HIF-1α and/or lox on BMP9-increased Wnt/β-catenin signaling activation

**Fig. 6:**
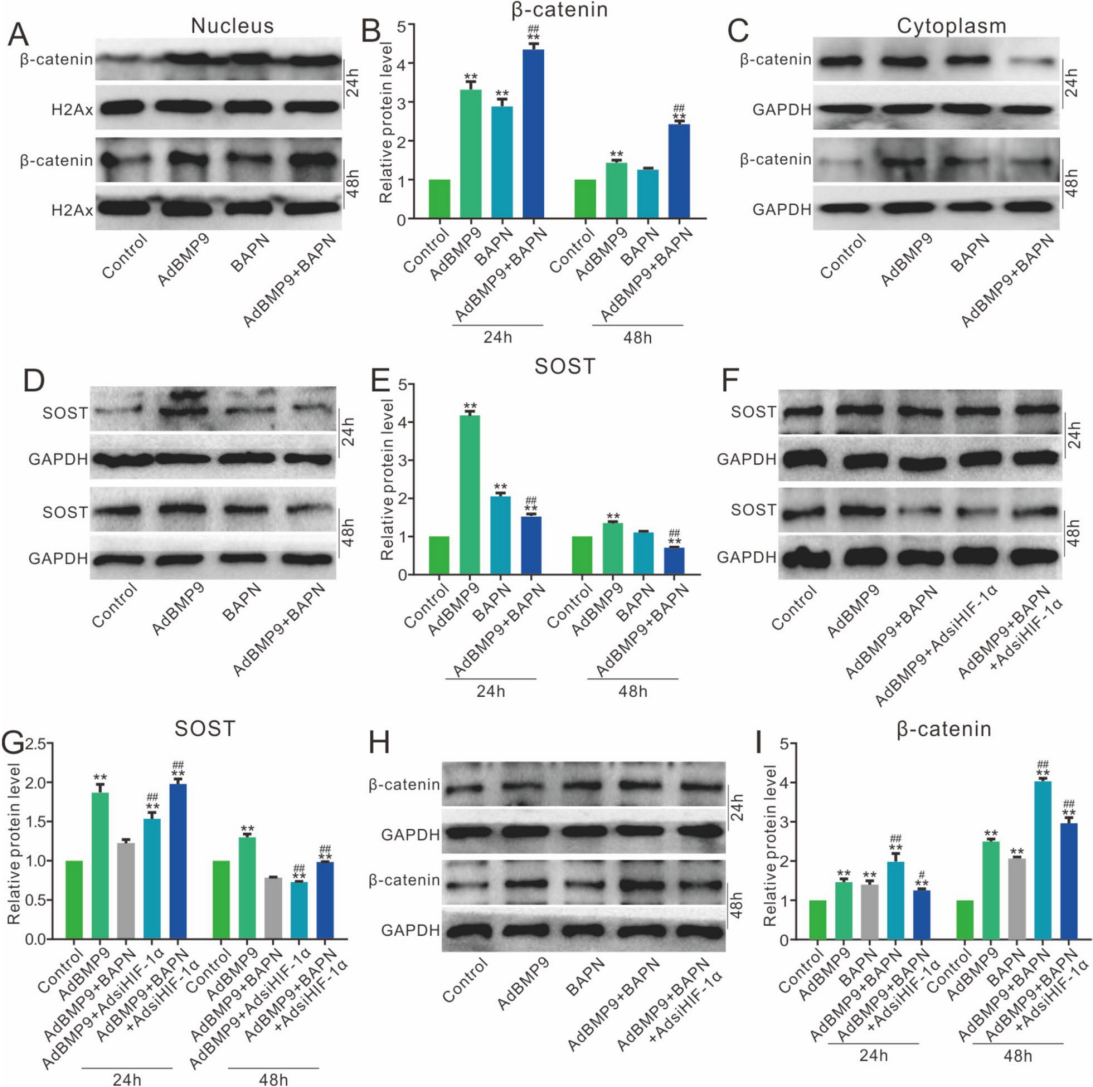
Lox and/or HIF-1α affects the effect of BMP9 on Wnt/β-catenin pathway **A**, **B** Effect of BAPN on the level of β-catenin in nucleus increased by BMP9. **C** Effect of BAPN on the level of β-catenin in cytoplasm increased by BMP9. **D**, **E** Effect of BAPN on the level of SOST induced by BMP9. **F**, **G** Effect of BAPN and/or siRNA of HIF-1α on the level of SOST induced by BMP9. **H**, **I** Effect of BAPN and/or siRNA of HIF-1α on the level of β-catenin induced by BMP9. (BAPN: β-aminopropionitrile, inhibitor of Lox, the concentration is 200 μM. **p < 0.01 vs. control; ##*p* < 0.01 vs. group treated with BMP9

Western blot assay showed that Lox inhibition increased the β-catenin protein level in nucleus and reduced in cytoplasm (Fig. 6A, B and C). BMP9 increased the sclerostin (SOST) protein level in 3T3-L1 cells, which was reduced by Lox inhibition (Fig. 6D and E). However, the decrease of BMP9-induced SOST by Lox inhibition was partially reversed by HIF-1α knockdown (Fig. 6F and G). Correspondingly, western blot assay showed the BMP9-increased β-catenin protein was elevated by Lox inhibition, which was notably reduced by HIF-1α knockdown (Fig. 6H and I). These data suggested that HIF-1α may partially mediate the effect of Lox knockdown on increasing Wnt/β-catenin signaling activity through SOST repression at least.

## Discussion

In this study, we demonstrated that BMP9 up-regulates Lox in pre-adipocyte. The osteoblastic-induction capability of BMP9 was enhanced by Lox inhibition or knockdown, but diminished by Lox overexpression. The effect of Lox inhibition or knockdown on the elevation of BMP9 osteogenic potential may be resulted from increasing Wnt/β-catenin signaling activation through HIF-1α up-regulation.

In skeletal development, adipogenic and osteogenic processes occur concomitantly although they exclude mutually. The equilibrium between adipogenic and osteoblastic differentiation is one critical step to keep bone homeostasis. Osteogenesis may depend on the cost of adipogenesis, and vice versa [19, 20]. Bone mesenchymal stem cells (BMSCs) can be directed to either adipocyte or osteocyte. Overproduction of adipocytes derived from BMSCs are one of the key pathomechanisms of osteoporosis [21]. PPARγ is a well-known adipogenic transcriptional regulator. Small compounds known as PPAR agonists, such as rosiglitazone and pioglitazone, have been utilized as effective treatments for diabetes mellitus [22]. However, one of the most notorious adverse effects of these drug is osteoporosis [23]. For this reason, PPARγ agonist usually prescribed for the treatment of diabetes mellitus together with bisphosphate. Recent studies have shown that BMP9 may be an excellent alternate for BMP2 or BMP7 to treat the related bone diseases because of the stronger osteogenic potential than BMP2 and BMP7 [24–26]. Additionally, BMP9 elevated PPARγ expression, while PPARγ knockdown boosted the BMP9 osteogenic potency [6]. However, the detailed mechanism is not fully elucidated.

The adipogenic commitment of stem cells are controlled by various factors, such as PPARγ, TRAF4, and Leptin [27–29]. To date, it remains unclear whether some certain factors exist that are exclusive to adipogenesis. It was reported that Lox plays an important role in adipogenesis during development [30], and aberrant Lox levels are associated with the biological behavior of cancer cells like metastasis [31]. Given this, Lox may also help stem cells maintain a balance between the processes of adipogenesis and osteogenesis. Lox can promote adipogenesis through inhibiting the FGF-2 signaling, or promoting the adipogenic transcriptional factors, such as PPARγ and CCAAT enhancer binding protein (C/EBP) α in 3T3-L1 cells [30]. Due to the mutual exclusion between osteogenesis and adipogenesis, Lox may also participate in regulating osteoblastic differentiation. Jover et al. reported that Lox over-expression promoted vascular smooth muscle cells calcification while Lox inhibition or knockdown decreased the calcification [31]. On the other hand, Lox inhibition greatly enhanced the BMP4-induced osteoblastic differentiation in MSCs [32]. The controversial effects of Lox on calcification or mineralization may contribute to the different of cell types, and context. In this study, we found that Lox inhibition or knockdown promoted the osteoblastic markers or bone masses induced by BMP9 in 3T3-L1 cells. These evidences supported that Lox may function as an important regulator for adipogenesis and osteogenesis, and its function may different greatly from other adipogenic factors like PPARγ.

An expanding evidences supported that Wnt/β-catenin can promote bone development or prevent osteoporosis by maintaining bone density [33, 34]. The osteogenic potential of BMP9 can be enhanced by Wnt/β-catenin, and reduced by β–catenin knockdown obviously in MSCs. Accordingly, BMP9 increased the Wnt/β-catenin signaling activity apparently [35]. All-trans-retinoic acid promoted the osteogenesis induced by BMP9 through increasing Wnt/β-catenin signaling activation [36]. However, it is unclear how BMP9 regulates Wnt/β-catenin signaling. Our data showed that the BMP9-induced osteoblastic markers and β-catenin level were increased by Lox inhibition or knockdown. Meanwhile, BMP9 increased the sclerostin (SOST), an Wnt/β-catenin inhibitor, yet it was abolished by Lox inhibition. These results implied that the effect of BMP9 on activating Wnt/β-catenin signaling may be mediated by Lox partially.

Hypoxia-inducible factor-1α (HIF-1α) regulates a variety of pathological processes, for example, it can facilitate the epithelial to mesenchymal transition through activating Lox in paraquat-induced pulmonary fibrosis [37]. HIF-1α and Lox were also regulated mutually to promote tumor cells growth [38]. It was reported that BMP9 upregulated HIF-1α to increase angiogenic signaling, which synergistically enhance its osteogenic capability [39]. Therefore, the regulatory effect of Lox on BMP9 osteogenic potential might be mediated by HIF-1α. We found that HIF-1α was increased by BMP9 in 3T3-L1 cells, which was enhanced by Lox inhibition or knockdown but reduced by Lox over-expression. HIF-1α can activate Wnt/β-catenin signaling via regulating BCL9 expression in hepatocellular carcinoma [40]. Hence, the effect of Lox on BMP9-increased Wnt/β-catenin signaling may be mediated through HIF-1α. Further analysis results exhibited that the SOST protein level was increased by BMP9, but reduced by Lox inhibition. Correspondingly, the total β-catenin protein level was elevated by BMP9 also. The increase of β-catenin by BMP9 was enhanced by Lox inhibition, but reduced notably by HIF-1α knockdown.

## Conclusion

Summary, our findings indicated that the Lox may decrease BMP9 osteogenic potential, which may be mediated by reducing Wnt/β-catenin signaling via HIF-1α/SOST pathway although the details need to be further investigated. Meanwhile, our finding also suggested that disturbing the Lox function may be another possible strategy to promote osteogenic differentiation of multipotent stem cells, which may accelerate bone tissue engineering development.

## Data Availability

The datasets generated and analyzed during the current study are available from the corresponding author on reasonable request.
